# Abandon “Race.” Focus on Racism

**DOI:** 10.3389/fpubh.2021.689462

**Published:** 2021-09-07

**Authors:** Paula Braveman, Tyan Parker Dominguez

**Affiliations:** ^1^Department of Family and Community Medicine, Center for Health Equity, University of California, San Francisco, San Francisco, CA, United States; ^2^Suzanne Dworak-Peck School of Social Work, University of Southern California, Los Angeles, CA, United States

**Keywords:** racism, race, racialization, racial classification, racial/ethnic classification, public health monitoring, public health research

## Abstract

The concept of “race” emerged in the 1600s with the trans-Atlantic slave trade, justifying slavery; it has been used to justify exploitation, denigration and decimation. Since then, despite contrary scientific evidence, a deeply-rooted belief has taken hold that “race,” indicated by, e.g., skin color or facial features, reflects fundamental biological differences. We propose that the term “race” be abandoned, substituting “ethnic group” while retaining “racism,” with the goal of dismantling it. Despite scientific consensus that “race” is a social construct, in official U.S. classifications, “Hispanic”/”Latino” is an “ethnicity” while African American/Black, American Indian/Alaska Native, Asian/Pacific Islander, and European American/White are “races.” There is no scientific basis for this. Each grouping reflects ancestry in a particular continent/region and shared history, e.g., the genocide and expropriation of Indigenous peoples, African Americans' enslavement, oppression and ongoing disenfranchisement, Latin America's Indigenous roots and colonization. Given migrations over millennia, each group reflects extensive genetic admixture across and within continents/regions. “Ethnicity” evokes social characteristics such as history, language, beliefs, customs. “Race” reinforces notions of inherent biological differences based on physical appearance. While not useful as a biological category, geographic ancestry is a key social category for monitoring and addressing health inequities because of racism's profound influence on health and well-being. We must continue to collect and analyze data on the population groups that have been racialized into socially constructed categories called “races.” We must not, however, continue to use that term; it is not the only obstacle to dismantling racism, but it is a significant one.

## Introduction and Overview

Racism is a public health crisis requiring bold action on many fronts. One of those fronts, perhaps surprisingly, is semantic and conceptual in nature; this does not, however, mean that the issue is unimportant or abstract. Language has power. Words can matter. Words have meaning that can inspire, promote, condone, justify, or inhibit actions. Words are tools we use to build or reinforce a shared understanding. Take the word “race,” for example. The concept of “race” emerged in the late seventeenth century, with the rise of the Transatlantic slave trade, and was used to justify slavery by regarding Africans as innately and biologically inferior (1–7). “Race,” as construed since then and now, refers to the classification of humans based on phenotype—observable physical differences—which are assumed to reflect inherent biological differences. “Race” has been used to justify the exploitation, denigration, and decimation of groups of people throughout our history. Even when those using the term underscore the distinction between phenotype and genotype (underlying genetic makeup), “race” still is and will continue to be implicitly conceptualized as biological. This paper's aim is to initiate discussion of what will undoubtedly be a controversial proposal: to abandon the term “race” as a way of categorizing humans, while retaining the term “racism” as a necessary tool for dismantling it.

Instead of “race” we propose that “ethnic group” or “ethnicity” be used to encompass what are now commonly referred to as “racial or ethnic groups,” an approach that has prevailed in much of Europe for decades. We propose continued use of the terms African American/Black, American Indian/Alaska Native, Asian American, European American/White, Latino/Hispanic, Native Hawaiian/Other Pacific Islander, but explicitly denoting them as ethnic groups, reflecting geographic origin and ancestry, not “races,” which intrinsically connotes biological differences. All of these terms denote geographic ancestry in a particular continent or other large region of the world and the shared history and language, beliefs, and/or customs that often accompany geographic ancestry. The concept of ethnic group or ethnicity has been criticized at times as too ill-defined and broad to be useful, given the diversity within each group;(8, 9), ideally we would monitor and study much smaller ethnic groupings according to clear, explicit criteria. These large geographic ancestry groupings are, however, at a minimum, critically important to continue to monitor and study, principally because of racism, which holds profound, which holds profound implications for health and well-being. These groupings reflect—albeit very roughly—how people are perceived and treated.

Using “ethnic groups” to encompass not only those groups that have traditionally been referred to as such, but also those now called “races” or “racial groups” is more consistent with science than the current approach. The scientific consensus about “race” today is that it is a fundamentally social rather than biological construct. The differences in superficial secondary characteristics such as skin color and hair texture across different “racial” groups do not correlate with underlying fundamental biological differences (10–13). Given human migrations over tens of thousands of years, each group defined by geographic ancestry reflects extensive genetic admixture across and within continents/regions. For example, Baharian et al. (14) estimated that African Americans today have approximately 16.7% European ancestry; Solovieff et al. (15) concluded that African Americans have “from 20 to 30% admixture with Europeans.”

Despite this knowledge, in official U.S. public health classifications, “Hispanic” or “Latino” is regarded as an ethnic group, whose members can be of any “race,” while African American/Black, American Indian/Alaska Native, Asian, Native Hawaiian/Pacific Islander, and European American/White are classified as “races.” There is no scientific basis for considering African Americans, Indigenous peoples, Asian Americans, Native Hawaiians/Pacific Islanders, and European Americans to be “races,” not ethnic groups, while viewing Hispanics/Latinos as an ethnic group but not a “race.” All of these groupings reflect geographic ancestry. The continued distinction between “race” and “ethnicity” only serves to underscore the implication that “race” reflects biological differences.

The continued use of the term “race” is by no means the only or even the principal obstacle to addressing racism. It is, however, a significant obstacle because it is irremediably imbued with scientifically unfounded but nevertheless tenacious notions of biological differences and hierarchy which have long served to justify exploitation and oppression. The use of “race,” even by those who abhor racism, tends to reinforce those notions. Reflecting commonly held beliefs, ethnicity is consistently defined as a social or cultural characteristic, while race is defined as biological and/or based on physical traits (which implicitly reflecting biological differences) (16–21).

We are not the first to propose abandoning use of the word “race” for classifying humans and substituting “ethnicity.” Ashley Montagu proposed substituting ethnicity for race in the field of physical anthropology in the 1940s (22). Bradby (9) argued that in the face of the dangers of using “race,” “the most helpful strategy is to reject the term “race,” but to retain the words ‘racism’ and ‘racialization’ and to use the term ‘ethnicity’.” Furthermore, as noted earlier, in much of Europe today the words “race” or “racial” rarely appear and official statistics do not report on “race.” The term “ethnicity” is used to encompass characteristics that in the United States would span both “race” and ethnicity (8, 23, 24). This well-established approach that has prevailed across multiple decades—abandoning the term “race” and substituting ethnicity—was adopted in Europe after World War II, in horrified reaction to the genocide of 6 million Jews, who the Nazis regarded as a “race” (25, 26). Unfortunately, when they ceased using the term “race,” a positive action, the Europeans also ceased any official collection of routine data on the groups formerly called “races;” they now lack crucial evidence for routine monitoring of racism and its social and health effects (27). This is an error we must not commit.

While not useful as a biological category, “race” as currently categorized—African American/Black, American Indian/Alaska Native, Asian, European American/White, Native Hawaiian/Pacific Islander, along with Latino/Hispanic “ethnicity”—is a vitally important social category for monitoring, understanding, and intervening on differences in health (28). This is true because of racism's profound influence on health and well-being. Each of these geographic groupings reflects ancestral origin in a particular continent or other large region of the globe. These geographic groupings are of great social—and therefore health—meaning and significance principally because they reflect differences in how people are perceived and treated both currently and historically. They reflect experiences of racism. They reflect shared history, e.g., the genocide and expropriation of the lands of Indigenous peoples; African Americans' enslavement, oppression and disenfranchisement under Jim Crow laws and ongoing violations of civil rights; and Latin America's Indigenous roots and colonization by Spain and Portugal.

Because of the profound impact of racism on health and well-being, we must continue to collect data on these socially constructed categories that have been called “races.” We should not, however, continue to unintentionally or intentionally *racialize people*—i.e., to regard people as if they represent fundamentally distinct groups—by using that term. In the next section, we further examine the concept of “race,” how it has been used, and the implications.

## The Concept of Race

Is there just one race—the human race? Or are there intrinsically different groups of humans who are biologically distinct from each other? Despite extensive scientific evidence to the contrary, there has been a long-standing, widely held, deeply rooted, and unfounded belief that “race,” as reflected by skin color, hair texture, facial features and other superficial secondary physical characteristics, reflects fundamental biological differences. That is a convenient idea to hold if one wants to justify treating some people as undeserving of equal rights, autonomy, and respect.

In colonial America, the notion of race arose and not coincidentally co-evolved with the emerging trans-Atlantic slave trade in the late 1600s; it was used to justify the enslavement and brutal treatment of Africans (1–7). Globally, the separation of groups into fundamentally different, “superior” and “inferior” races has been foundational to justify both slavery and genocide (29). These notions also have been used to justify discriminatory laws, policies, and practices that deny equal rights and opportunities based on “race.” These discriminatory structures include the “Jim Crow” laws in formerly Confederate states that prevailed for almost 100 years after legal slavery officially ended (1, 30). They also include the lesser-known but also long-standing “Black Laws” denying the rights of Black people in Northern states, which predated and served as a model for Jim Crow laws (31). Pro-slavery doctors used pseudoscience to explain Black-White differences in anatomy and disease as innate and evidence of Black inferiority. Pro-slavery politicians amplified this pseudoscience to argue against abolition (4, 32).

Reflecting the belief in an underlying biological basis for “race,” official birth and death records and other health data in the United States have long been reported separately by age, sex, and “race.” Because age and sex do indeed reflect fundamental biological differences, this reporting practice implicitly tends to reinforce the erroneous notion of “race” as a biological construct. While changing “race” to “ethnic group” would not eliminate this conceptual problem, it may be a small but important step in helping us to reconceptualize superficial physical difference as reflective of social and cultural diversity rather than biology.

If race were biologically based, one might expect to find consistency in classification and consensus about some standard set of mutually exclusive racial groups. The concept of race has been very fluid, however, and operationalized in different ways at different times, which in itself suggests its fundamentally socially constructed nature. For example, the Nazi movement in twentieth century Germany regarded Jewish people as a “race;” the rationale for their mass extermination was that they were an inferior “race” that should be eliminated to avoid contaminating the superior race, Aryans (29). In the United States during the late nineteenth/early twentieth centuries, many economically struggling White people felt threatened by an influx of immigrants willing to work for lower wages. Immigrants from Ireland, Italy, and Poland were widely regarded as being from “races” distinct from and inferior to “Whites,” i.e., the more established European immigrants (33, 34). The more established immigrants asserted their group's superiority, beginning to express political power through various institutions (e.g., labor unions) and actions (e.g., race riots). Over time, the ethnic identities of the more recently arrived European immigrants were subsumed into “Whiteness,” and members of these groups came to benefit from the legal, political, and social advantages enjoyed by White people in the United States (33, 34).

Reflecting changing social, economic, and political forces over time in the United States, “races” have been officially categorized in various ways. The earliest U.S. Census surveys distinguished only Whites, all other free persons, and slaves. Later Census racial categories included White (European ancestry), Black (African ancestry), or Mulatto (mixed), and, only in the 1890 Census, “Quadroon” and “Octoroon,” classifying people by their percentage of African ancestry. Census racial categories have changed and expanded to reflect emancipation, immigration, social movements, and political pressure (35, 36). Currently, the established “racial” categories for reporting federal statistics in the U.S. are African American/Black, American Indian/Alaska Native, Asian, Native Hawaiian or Other Pacific Islander, and European American/White. Each of these groupings corresponds to ancestry in a continent or other large geographic region (37).

Since 1980, the U.S. Census and vital statistics have collected data on a separate field in addition to “race,” to specify whether a person is of “Hispanic origin.” This information is officially referred to as a measure of Hispanic *ethnicity* and not of race (38, 39). This implies that while African Americans, Asian Americans, European Americans (Whites), and Indigenous Americans each constitute a separate race, Latinos or Hispanics do not, they are an “ethnic group.” This classification implicitly reinforces a notion of race as biological, in contrast to ethnicity, which is social. Based on recognition that these two categories are not distinct, the term “race/ethnicity” is often used instead of either term alone. In practice, health research and reporting frequently classify people into five mutually exclusive categories referred to as “racial/ethnic” groups, based on the continent or other large geographic region of their ancestry. This is often done by creating a category for Latinos/Hispanics regardless of “race” and restricting each of the “racial” groups to non-Latinos/Hispanics in each of those groups. This widespread practice reflects the lack of conceptual coherence of the distinction between “race” and “ethnic group. “Latino/Hispanic” denotes Latin American origin/ancestry (“Latino” is arguably a more appropriate term than “Hispanic,” given that many Latin Americans—for example, those from Brazil, Latin America's largest country—are not Spanish-speaking, and people from Spain—who one would expect to be included as “Hispanics”—are Europeans).

Another example of the fundamentally social nature of “racial” classifications comes from a study examining the health advantage of Whiteness. Jones et al. (40) studied how self-reported health varied depending on whether it was examined in relation to survey respondents' “socially assigned race,” meaning, the “race” that other people generally assumed them to be, or the respondents' self-identified “race.” They found considerable differences in health based on self-identified vs. socially assigned “race.” The investigators found that, among respondents who self-identified as Black, Hispanic, or multi-racial, the prevalence of excellent or good self-reported health was significantly higher among those who were perceived by others to be White than among those of the same self-identified “race” who were not perceived by others to be White; the same pattern was seen for American Indians but was not statistically significant. A 2020 review of 18 studies of associations between a range of health indicators and socially assigned “race” reported that most studies have found an association between socially assigned “race” and health (41). These research findings, along with a large body of other accumulated knowledge, support the realization that it is the social experience of living in bodies perceived to be of different “races,” which society treats differently, rather than fundamental biological differences between people of different “races,” that generally drives differential health outcomes.

Studies of human genetics have shown repeatedly that there is more genetic variation among people with the same geographic ancestry than there is between groups of people with different geographic ancestry (42). The scientific consensus about racial categories today is that they are fundamentally social rather than biological constructs (10–13). Craig Venter, head of Celera, the private genetics company that partnered with the National Institutes of Health on the Human Genome Project, stated in a White House briefing on the Human Genome Project in June of 2000 (and note the use of the term “ethnicity” vs. “race”):

“The method used by Celera has determined the genetic code of five individuals. We have sequenced the genome of three females and two males, who have identified themselves as Hispanic, Asian, Caucasian or African American. We did this sampling not in an exclusionary way, but out of respect for the diversity that is America, and to help illustrate that the concept of race has no genetic or scientific basis. In the five Celera genomes, there is no way to tell one ethnicity from another.”

This does not imply that there cannot be any genetic differences among people in groups with different geographic ancestry. Rather, it means that differences in superficial secondary physical characteristics such as skin color, facial features, or hair texture do not define biologically distinct groups. Neither do differences in some gene frequencies, nor any of the isolated, highly specific genetic differences that have been found across different geographic ancestry groups define biologically distinct groups. The racialization of sickle cell disease is an instructive example. If a person inherits the sickle cell mutation from both parents, sickle-shaped (deformed) hemoglobin is produced, which, when exposed to low levels of oxygen, damages red blood cells, leading to “acute vasoocclusive events, hemolytic anemia, organ damage and failure, and an average lifespan reduction in developed countries of more than three decades” (15). While the mutation is most common among people with ancestry in three areas of West Africa, it is not common among people from other areas of Africa, furthermore, it is also seen among people with Mediterranean, Middle Eastern or Indian ancestry, regions which, like West Africa had a high prevalence of malaria. The mutation came to be frequent in those regions because it was protective against malaria, giving an evolutionary advantage to carriers (people with only one affected gene). This highly specific genetic difference among African Americans from three areas of West Africa and people from the Mediterranean, Middle East, and India, compared to most Africans and Europeans arose because of evolutionary pressures. It does not occur among African Americans from most parts of Africa—only those from specific areas in West Africa, and it occurs among people with non-African ancestry. Despite these undisputed facts, sickle cell disease has been racialized; this isolated genetic difference does not define a “race.” Another example is Tay-Sachs disease among Jewish people of Northern European ancestry, the greater frequency of the mutation that produces Tay-Sachs disease does not make Jewish people a biologically distinct group.

It should be noted, furthermore, that even when there are isolated genetic differences between groups of people with different geographic ancestry, those differences may not be expressed (i.e., the genes may not have their potential effects on the body) unless people are exposed to certain environmental influences, including stressors, which are shaped by social forces. Social experiences often control whether or not genetic differences are expressed or suppressed, this underscores the social nature of “racial” categories. The next section discusses how racism affects health.

## Racism and Its Effects on Health

Racism is a fundamental social determinant of racial disparities in health (43). It is a system of “race”-based power, rooted in notions of inherent racial group superiority and inferiority, that systematically, pervasively, and unjustly privileges “Whites” and oppresses “non-Whites” (44). It manifests at multiple levels (internalized, interpersonal, institutional, and structural or systemic) and involves direct, indirect, individual, and group-based exposures across the life course (45, 46). Whether blatant, subtle, or ambiguous, racism is a particularly visceral threat to well-being because it denigrates a core aspect of a person's identity that is ever-visible and unchanging.

Racism produces racial discrimination, i.e., “race”-based unfair treatment. Although racial discrimination is no longer legal, socioeconomic, and health inequities along racial lines persist because of deeply rooted, unfair systems and structures that continue to operate to sustain the legacy of formerly overtly discriminatory practices, policies, laws, and beliefs. At times, these systems and structures operate unintentionally, but nevertheless effectively, to produce and sustain racial discrimination.

An extensive body of literature demonstrates large and pervasive disparities in health adversely affecting African Americans/Black people (47, 48), American Indians/Alaska Natives (49), Hispanics/Latinos (50), some Asian subgroups (51), Native Hawaiians/Pacific Islanders (52–54), and immigrants compared to Whites (47, 48, 51, 55). These differences are seen for a wide range of health indicators and across the lifespan (56–59). The largest and most consistent racial/ethnic health disparities generally have been observed when comparing Black people and (when data are available) American Indians with Whites, although Latinos and some Asian/Pacific Islander groups have worse health than Whites on some measures. What explains these pervasively and repeatedly observed differences in health across a wide array of health indicators?

Racism can damage the health of people of color through many different causal pathways. An extensive and growing body of scientific research indicates how diverse experiences of racism likely play a fundamental role in producing racial/ethnic inequities in health by activating multiple causal pathways. Some of these pathways are complex and long, often playing out over lifetimes and even generations. The complexity and length of the causal pathways often makes it difficult to detect their origins, that is, the underlying but hidden fundamental causes. The term “embodiment” has been used by several scholars within and outside the health field to describe how racism in its myriad forms ultimately produces damages to health, which are then misinterpreted as signs of underlying biological differences among racialized groups (60, 61).

Although the focus here is on how racism can damage the health of people of color, it is important to note that racism is likely to damage the health and well-being of virtually the entire society in which it operates. The support for this hypothesis comes from research on social inequality in general rather than on racism specifically: A convincing case has been made that social inequality damages the health of societies overall (62, 63), largely by undermining social cohesion (64).

Based on the literature, the figure (above) depicts a series of sequential general steps through which racism is thought to produce racial disparities in health. These general steps represent the basic skeleton of innumerable specific pathways, which are indicated by the factors listed in each box as non-exhaustive) examples.

**Figure 1:**
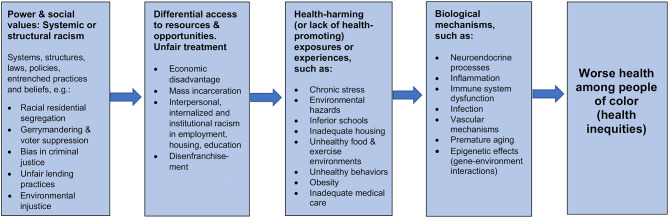
How racism is thought to damage health: a general overview of key sequential steps.

- The first box on the left in Figure 1 represents the beginning of the general sequence. The common beginning or source of all the potential causal pathways is systemic or structural racism, the unjust systems or structures that systematically put people of color at a disadvantage in multiple domains. Systemic or structural racism reflect underlying differences in power and social values in a society. The relevant systems or structures include laws, policies (written and unwritten), entrenched institutional practices, and the pervasive, deeply rooted beliefs that condone, promote, and perpetuate the systems and structures. Examples of systemic or structural racism in the past include: slavery and the laws and beliefs that supported it; Jim Crow laws and the systematic use of terror to enforce them; and redlining and the discriminatory implementation of the G.I. Bill and low-interest FHA loans that enabled many White people of modest means to become homeowners but were given to very few Blacks (65, 66). These historical systems and structures set the stage, establishing the inequitable patterns and providing a firm basis for their perpetuation including: disenfranchisement via gerrymandering and voter suppression; racial residential segregation; unfair lending practices depriving people of color of the opportunity to own a home or to start or expand a business, thereby closing off opportunities to accumulate wealth; the dependence of schools on local property taxes, ensuring that schools in areas that lack wealth are under-resourced; and mass incarceration of men of color based on biased policing and sentencing.
- The 2nd box from the left in Figure 1 represents the next general step in the sequence: the direct products of systemic or structural racism which are unfair treatment and differential access to resources and opportunities. Arguably, the borders between this 1st box on the left and the 2nd box should be depicted as porous, as many examples of systemic or structural racism not only produce but in themselves constitute unfair treatment and lack of access to resources and opportunities. Examples of unfair treatment and lack of access to resources and opportunities that are generally due to systemic or structural racism include: both interpersonal and internalized racism; less favorable treatment in hiring, retention, promotion, and pay; the socioeconomic disadvantage that results from being trapped in low-opportunity segregated areas with substandard schools, poor services, and scant hope of escaping unrelenting financial hardship; and the permanent stigmatization of people who have been incarcerated, permanently blocking ex-prisoners from employment opportunities, thus ensuring ongoing financial hardship for the ex-prisoners, their families, and communities.
- As depicted in the third box from the left, the unfair treatment and differential access to resources and opportunities result not only in exposure to health-harming conditions (such as toxic environmental hazards and chronic stress), but also limited access to conditions that are health-promoting, such as good schools, a nutritious diet, green spaces, bicycle lanes, being able to afford a gym membership, and quality medical care.
- These health-harming (or lack of health-promoting) exposures or experiences in turn trigger biological mechanisms that produce ill health (the fourth box). These biological mechanisms include, for example, inflammation and alterations in the functioning of the immune system that are known to lead to chronic diseases, such as heart disease, stroke, and diabetes. The biological mechanisms include neuroendocrine processes triggered by chronically stressful experiences (such as experiencing discrimination or persistent financial hardship). These neuroendocrine processes result in the body's production of hormones (like cortisol and other substances) that can, if present at persistently high levels over time, lead to inflammation and dysfunction of the immune system, both of which contribute to susceptibility to chronic disease and premature aging (67).

This section of the paper has sought to demonstrate that, although further research is needed, there is now considerable evidence of how racism sets in motion a range of phenomena that damage health in multiple domains. The damage is inflicted by unfair treatment and differential access to resources and opportunities, which produce health-harming exposures (and lack of health-promoting experiences), which in turn trigger an array of physiologic processes that directly produce ill health. Racial differences in health overwhelmingly reflect racism.

## Concluding Remarks

The concept of “race” had its origins in the slave trade. It is time to abandon it. The concept was used to justify slavery by implying that those who were enslaved were fundamentally different and inferior beings. Today “race” continues to reinforce false and weaponized notions of inherent biological differences based on physical appearance. It should be used rarely and only inside quotation marks when necessary to make a point about its historical usage. “Ethnic group” or “ethnicity” should be substituted, consistent with the social characteristics that often accompany geographic ancestry. The feasibility of using “ethnic group” or “ethnicity” in place of “race” is suggested by the fact that this has been standard practice in Europe since soon after the end of World War II. Has the fact that Europe has largely substituted ethnic group for race ended racism there? Of course not. But it does mean that racism is not being perpetually and unnecessarily reinforced by the use of a term that inevitably evokes biological difference. Wherever this approach is adopted, if it is, vigilance will be needed to detect and halt conscious or unconscious attempts to redefine ethnicity as biological. Abandoning “race” will not in itself end racism or even help us to focus more on racism; we have called for focusing on racism to make it clear that abandoning the term “race” is part of a struggle against racism; and that the term “racism” must be retained. Abandoning “race” should, however, remove one ubiquitous and not inconsequential source of constant reinforcement of racism. Abandoning “race” should be part—admittedly a small part—of a strategy to intensify and broaden actions against racism. While abandoning “race,” we should not only retain the term “racism” but mount more intensive efforts to identify and dismantle it. In particular, we need to focus on systemic or structural racism, often invisible but posing the greatest barriers to justice and health because it is the root source of the varied manifestations of racial discrimination observed in multiple domains.

While not useful as a biological category, geographic ancestry—i.e., African American, American Indian/Alaska Native, Asian, Native Hawaiian/Pacific Islander, European American, and Latino/Hispanic—is a key social classification for monitoring and addressing health inequities. These geographic ancestry groups are very broad, with great heterogeneity within each. To address this, we ideally would monitor and study much smaller and better-defined ethnic groups. Because of racism's profound influence on health and well-being, however, we must, at a minimum, continue to collect and analyze data on the population groups that have been racialized into socially constructed categories called “races.” It is unfortunate that in Europe, abandoning the term “race” has not been accompanied by routine monitoring of health and well-being according to markers of the ethnic groups that are relevant to racism. We must continue to monitor differences in health and well-being according to geographic ancestry. We must not, however, continue to use the term “race.” It is not the only obstacle to dismantling racism, but it is a significant one, one that amplifies the damage every time it is used, even when used by those who actively struggle against racism. It is beyond the scope of this paper to discuss the implications of the use of “Black” and “White” in ongoing population monitoring and research, important and complex issues are involved there as well.

Bhopal (16) offered the following statement “for reflection and debate”: “The term “race” should be used with caution for its history is one of misuse and injustice.” Like Bhopal, we offer these thoughts to stimulate reflection and debate; however, we advocate not merely using “race” with caution but abandoning it entirely because it inherently lends itself to perpetuating falsehoods that promote serious harm and injustice. The public health crisis produced by racism requires fighting vigorously in many arenas, including conceptual and semantic spheres. Abandoning “race” is not a panacea but may be a useful adjunct to other crucial efforts to dismantle racism in all of its forms.

## Author Contributions

PB wrote the first draft after discussion with TP. TP added substantially to that and made revisions. All authors consulted closely with each other and participated in final revisions.

## Conflict of Interest

The authors declare that the research was conducted in the absence of any commercial or financial relationships that could be construed as a potential conflict of interest.

## Publisher's Note

All claims expressed in this article are solely those of the authors and do not necessarily represent those of their affiliated organizations, or those of the publisher, the editors and the reviewers. Any product that may be evaluated in this article, or claim that may be made by its manufacturer, is not guaranteed or endorsed by the publisher.
